# A novel 3’,5’-diprenylated chalcone induces concurrent apoptosis and GSDME-dependent pyroptosis through activating PKCδ/JNK signal in prostate cancer

**DOI:** 10.18632/aging.103178

**Published:** 2020-05-19

**Authors:** Yongqiang Zhang, Jue Yang, Zhonghang Wen, Xiaoyue Chen, Jia Yu, Dongbo Yuan, Bixue Xu, Heng Luo, Jianguo Zhu

**Affiliations:** 1 State Key Laboratory of Functions and Applications of Medicinal Plants, Guizhou Medical University, Guiyang 550014, P.R. China; 2 Guizhou Provincial People’s Hospital, Guiyang 550002, P.R. China; 3 Key Laboratory of Chemistry for Natural Products of Guizhou Province and Chinese Academy of Sciences, Guiyang 550014, P.R. China

**Keywords:** 3’,5’-diprenylated chalcone, PKCδ, apoptosis, pyroptosis, crosstalk

## Abstract

Although androgen deprivation therapy may initially be effective in prostate cancer, the disease can gradually progress to castration-resistant prostate cancer, at which point chemotherapy becomes the major clinical strategy. In this study, we demonstrated the anti-cancer potential of a novel 3’,5’-diprenylated chalcone (C10), which selectively inhibited the proliferation of PC3 cells *in vitro* and *in vivo*. C10 treatment elevated the proportion of PC3 cells in sub-G1 phase and induced programmed cell death. Interestingly, C10 elicited concurrent Caspase-dependent apoptotic and gasdermin E-dependent pyroptotic events. RNA-Seq and bioinformatics analyses revealed a strong correlation between protein kinase C delta (PKCδ) and mitogen-activated protein kinase pathway activation in prostate cancer. PKCδ silencing in PC3 cells suppressed the activation of the JNK pathway and the expression of its downstream genes, including Bax, interleukin-6 and interleukin-1β, which are involved in apoptotic and pyroptotic processes. Moreover, in PC3 cell xenograft tumor tissues, C10 treatment inhibited tumor growth and upregulated PKCδ. These findings suggest that C10 treatment induces the PKCδ/JNK pathway, thereby activating Caspase-3 and inducing the cleavage of PARP and gasdermin E to execute apoptosis and cell-lytic pyroptosis in prostate cancer cells.

## INTRODUCTION

Prostate cancer (PCa) is the most common solid organ malignancy and the second leading cause of cancer-related death in men worldwide [1]. The majority of new presentations involve locally advanced disease, representing a significant healthcare issue and economic burden. Androgen deprivation therapy is widely recognized as the most effective palliative treatment for non-advanced patients [2, 3]. However, most patients receiving androgen deprivation therapy will progress to castration-resistant PCa within 14-20 months; thus, chemotherapy combined with radiotherapy has become a major clinical strategy for advanced PCa [4].

Programmed cell death (PCD) pathways including apoptosis, necrosis, necroptosis, pyroptosis and others are physiologically crucial for the growth, survival and innate immunity of all multicellular organisms [5–7]. Apoptosis is the traditional mode of PCD, and can occur by two distinct signaling cascades: the extrinsic and intrinsic pathways [7]. Both pathways depend on the activation of cysteine proteases, termed Caspases, which ultimately trigger cell death characterized by cellular shrinkage, chromatin condensation and membrane blebbing [8–10]. The extrinsic pathway requires the recruitment of the adaptor protein Fas-associated death domain to activate Caspase-8 [11]. In contrast, the intrinsic pathway is activated by mitochondrial damage, which is followed by the release of cytochrome C, apoptosis-inducing factor and pro-Caspase-9 into the cytosol and the formation of a complex termed the apoptosome [12, 13]. Notably, apoptotic bodies develop in homeostatic environments and are rapidly phagocytosed by scavenger cells without an inflammatory response [14].

However, chemotherapy drugs induce both apoptotic and pyroptotic death, and the major distinction between these two kinds of PCD is their immunologic outcomes. Programmed pyroptosis, an inflammatory form of PCD, is characterized by cellular swelling, cell lysis and inflammatory cytokine release [15, 16]. Pyroptosis is driven by two significant signaling pathways – the canonical inflammatory Caspase-1/4/5 pathway (orthologous to Caspase-11 in mice) and the non-canonical Caspase-3 pathway – and is followed by the release of inflammatory factors such as interleukin (IL)-1β and IL-18 into the extracellular environment [17–20]. If the inflammasome is sufficiently stimulated, activated Caspase-1/4/5/11 will cleave the hinge region of gasdermin D (GSDMD) to generate C-terminal and N-terminal fragments after Asp275. GSDMD-N will then translocate to and perforate the membrane. Gasdermin E (GSDME), a GSDMD-related family member, is a physiological substrate for activated Caspase-3, which generates a necrotic N-terminal GSDME fragment capable of inducing pyroptosis [20, 21]. Notably, among human Caspases, only Caspase-3 is able to efficiently cleave GSDME after Asp270 [5].

The mammalian protein kinase C (PKC) family consists of phospholipid-dependent serine/threonine kinases that have been definitively classified into three subfamilies according to their dependence on the second messengers diacylglycerol and calcium: “conventional/classical” cPKCs (α, βI, βII and γ), “novel” nPKCs (δ, ε, η and θ) and “atypical” PKCs [22]. PKCs principally participate in transmembrane signal transduction pathways and biological processes contributing to cell proliferation, apoptosis, inflammation and drug resistance [23–25]. The delta isoform (PKCδ) is a critical upstream regulatory factor that is involved in several diseases, including cancer and neurodegenerative diseases [26, 27]. Previous studies from our lab and others have demonstrated that PKCδ phosphorylation and mitogen-activated protein kinase (MAPK) pathway activation can induce erythroleukemic cell differentiation [28].

**Figure 1:**
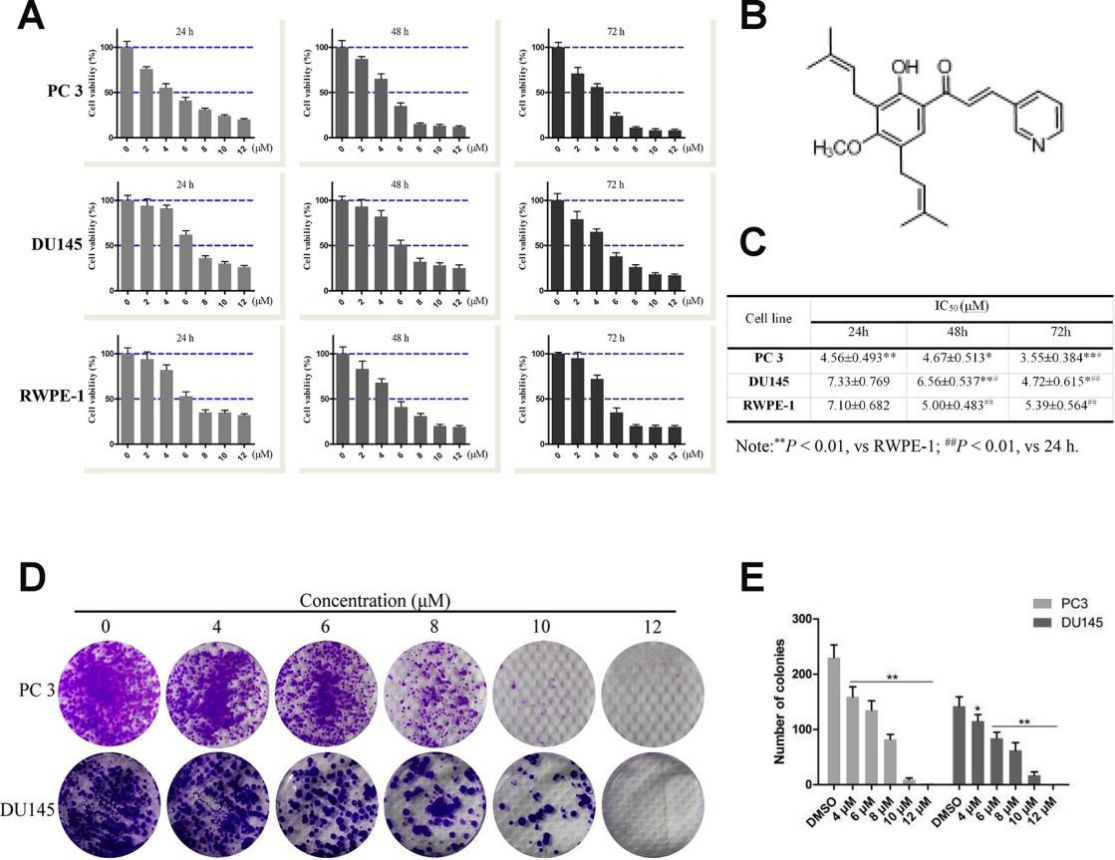
**Effects of compound 10 on PCa cell viability and proliferation.** (**A**) PC3, DU145 and RWPE-1 cells were treated with various concentrations of C10 for 24, 48 or 72 h, and cell viability was analyzed with an MTT assay. (**B**) Chemical structure of flavagline-like compound 10. (**C**) The IC_50_ values (μM) of the indicated cell lines were measured at three time points (24, 48 and 72 h) and summarized in a table. (**D**, **E**) C10 significantly inhibited the proliferation of PC3 and DU145 cells. A clonogenic assay was performed, and the number of colonies formed was analyzed. Per condition, three independent experiments were performed. Data are shown as the mean ± SD, *P < 0.05, **P < 0.01 vs. the control group.

The paradigm of oncology research is shifting toward targeted approaches that alter important molecular pathways for cancer prevention and therapy. Chalcones, due to their structural diversity, have various biological activities, including anti-inflammatory, anti-cancer and anti-mutagenic functions [29]. We previously designed and synthesized a series of novel 3’,5’-diprenylated chalcones and screened them for anti-cancer activity [30]. In the present study, we found that one of these chalcones (C10; structure shown in Figure 1B) triggered concurrent apoptosis and GSDME-dependent pyroptosis in PCa cells by activating PKCδ/JNK signaling.

## RESULTS

### Effects of C10 on the viability and proliferation of RWPE-1, DU145 and PC3 cells *in vitro*

The 3-(4,5-dimethyl-2-thiazolyl)-2,5-diphenyl-2-H-tetrazolium bromide (MTT) assay was used to assess the effects of C10 on the viability of normal prostate (RWPE-1) and prostate cancer (DU145 and PC3) cells (Figure 1). In all cell types, treatment with increasing concentrations of C10 (0-12 μM) for 24, 48 or 72 h (vs. 0.1% DMSO as a vehicle control) dose-dependently and time-dependently reduced cell viability (Figure 1A). At each treatment time, PC3 cells (IC_50_: 4.56±0.493, 4.67±0.513 and 3.55±0.384 μM, respectively) were more sensitive to C10 treatment than DU145 and RWPE-1 cells (Figure 1C).

Next, a clonogenic assay was used to further assess the effects of C10 on PC3 and DU145 cell proliferation. Increasing concentrations of C10 (0-12 μM) significantly reduced the proliferation and colony formation abilities of the cells simultaneously. After 6 μM C10 treatment, the colony formation efficiencies of the two cell types differed significantly, demonstrating that C10 may exert better inhibitory effects on PC3 cells than on DU145 cells (Figure 1D and 1E).

### Effects of C10 on overall transcriptomic changes and signaling pathways in RNA-Seq analysis

**Figure 2:**
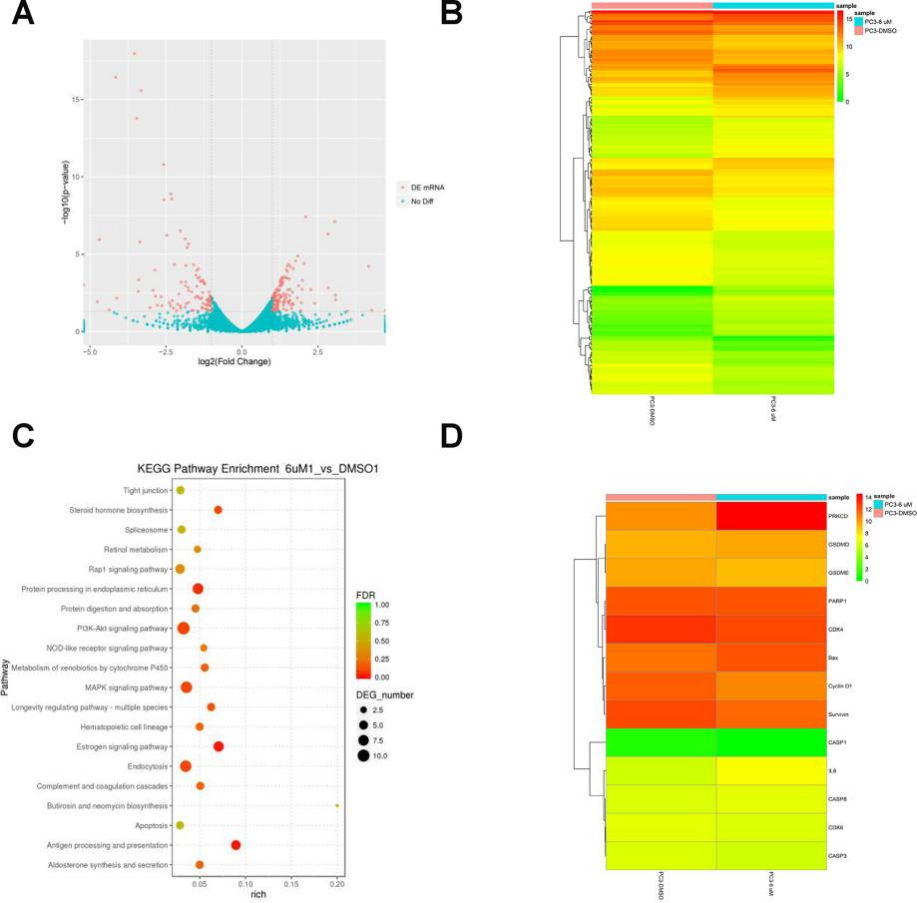
**RNA-Seq analysis of overall transcriptomic changes in C10-treated PC3 cells.** (**A**) The differentially expressed genes were redacted and visualized as a volcano plot. The red dots represent significantly differentially expressed genes, while the blue dots indicate non-significantly differentially expressed genes. (**B**) Heatmap of genes upregulated or downregulated by C10 treatment in PC3 cells. (**C**) KEGG enrichment analysis of signaling pathways altered by C10 treatment. The color and size of the dots indicate the significance of the false discovery rate and the number of differentially expressed genes in the pathway, respectively. The top 20 significantly enriched signaling pathways were profiled. (**D**) The dysregulated genes involved in the cell cycle, apoptosis and pyroptosis were screened from all the raw data, compiled into a new list and shown as a heatmap.

To investigate the molecular mechanisms involved in the proliferation and PCD of PCa cells, we performed mRNA sequencing on vehicle- and C10-treated PC3 cells. Differentially expressed genes were identified as those exhibiting transcriptional |fold change| values > 2 and *P*-values < 0.05. After C10 treatment, the cells displayed an anti-PCa transcriptional signature defined by 237 differentially expressed genes, including 136 upregulated and 101 downregulated genes. The dysregulated genes were redacted using the R package ggplots2 software and depicted as a volcano plot (Figure 2A). Genes that were consistently significantly changed in the two samples were compiled and visualized as a heatmap (Figure 2B and 2D).

Subsequent Kyoto Encyclopedia of Genes and Genomes (KEGG) enrichment analyses revealed that the differentially expressed genes were enriched in pathways associated with the cell cycle, apoptosis, MAPK and nucleotide-binding oligomerization domain (NOD)-like receptor signaling (Figure 2C, Supplementary Table 1). Interestingly, the mRNA levels of PKCδ, Bax and IL-6 were all upregulated in C10-treated cells, while there were no obvious changes in Caspase-3 or Caspase-8 levels, and Caspase-1 was not detected. Previous studies have demonstrated that PKCδ promotes apoptosis by stimulating the MAPK pathway and inflammatory infiltration [26, 28]. Thus, C10 may arrest the cell cycle, promote apoptosis and induce secondary pyroptosis in PC3 cells.

### Effects of C10 on cell cycle progression and cell cycle regulatory gene expression in PC3 cells

**Figure 3:**
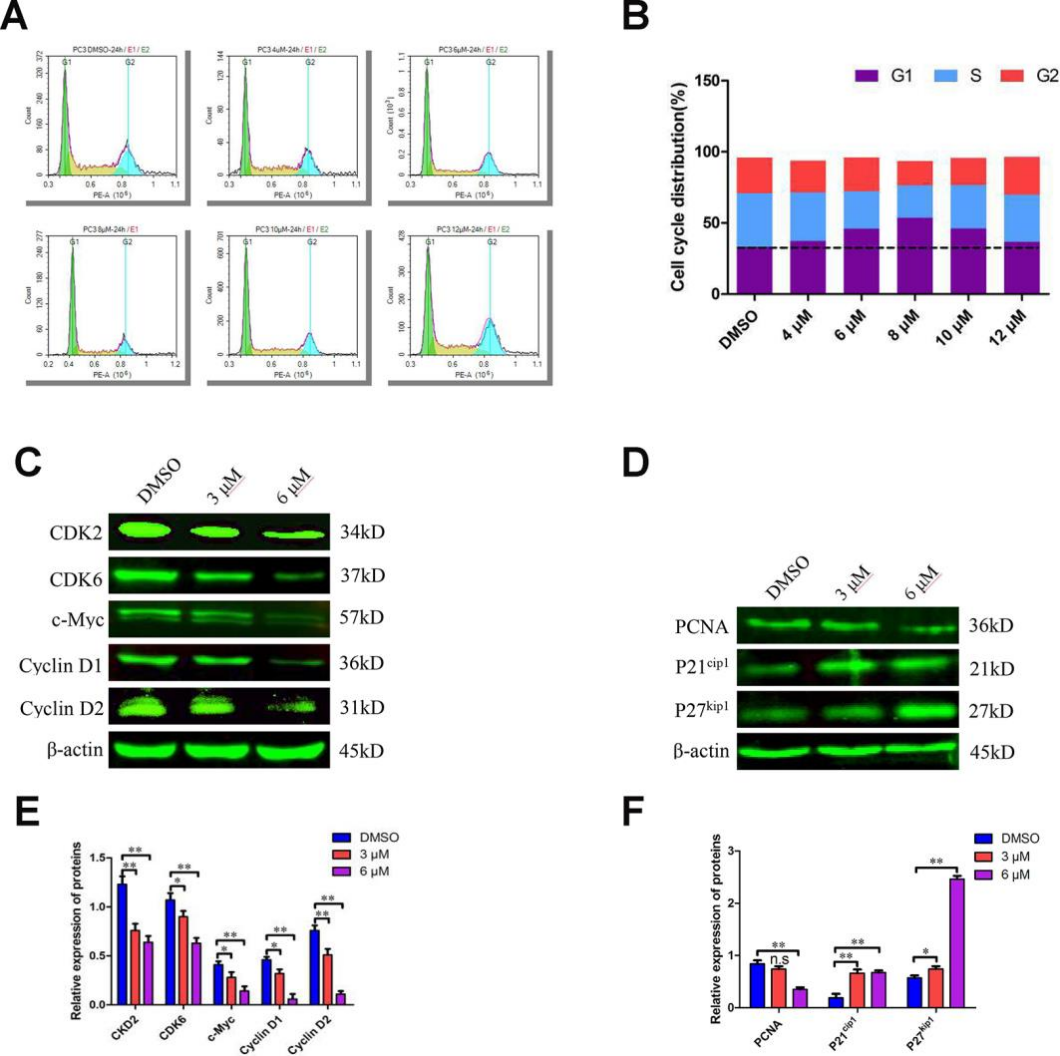
**C10 induced sub-G1 phase arrest of PC3 cells in culture.** (**A**, **B**) PC3 cells were treated with C10 (0, 4, 6, 8, 10 or 12 μM) for 24 h and stained with PI so that the DNA content could be analyzed by flow cytometry. C10 increased the proportion of PC3 cells in sub-G1 phase of the cell cycle. (**C**–**F**) Western blots of PC3 cells treated for 24 h with C10. The blots were probed with antibodies against CDK2, CDK6, c-Myc, Cyclin D1, Cyclin D2, PCNA, P21^cip1^ and P27^kip1^. All data shown are representative of three independent experiments. Data are shown as the mean ± SD. *P < 0.05, **P < 0.01 vs. the control group.

Since C10 inhibited PCa cell proliferation, we further examined its effects on cell cycle progression. Treatment of PC3 cells with various concentrations of C10 (0, 4, 6 and 8 μM) for 24 h dose-dependently arrested the cell cycle at sub-G1 phase; thus, at the 8 μM dose, 53.50% of the C10-treated cells exhibited sub-G1 phase arrest, versus 33.18% of the control cells. However, at higher doses of C10 (10 and 12 μM), the proportion of PC3 cells in sub-G1 phase decreased significantly, indicating that DNA fragmentation and necrosis had occurred (Figure 3A and 3B).

To understand the underlying molecular events, we examined numerous cell cycle regulators. Both quantitative real-time PCR (qRT-PCR) and Western blot analyses revealed that CDK2, CDK6, Cyclin D1, Cyclin D2 and c-Myc levels were consistently reduced in C10-treated cells, whereas P21^cip1^ and P27^kip1^ levels were substantially elevated (Figure 3C–3F, Supplementary Figure 1). In addition, proliferating cell nuclear antigen (PCNA), a downstream marker of proliferation, was remarkably downregulated in C10-treated cells. Thus, C10 arrested growth in sub-G1 phase by upregulating P21^cip1^ and P27^kip1^ and downregulating CDKs and Cyclins.

### Effects of C10 on cell death via Caspase-dependent apoptotic events

**Figure 4:**
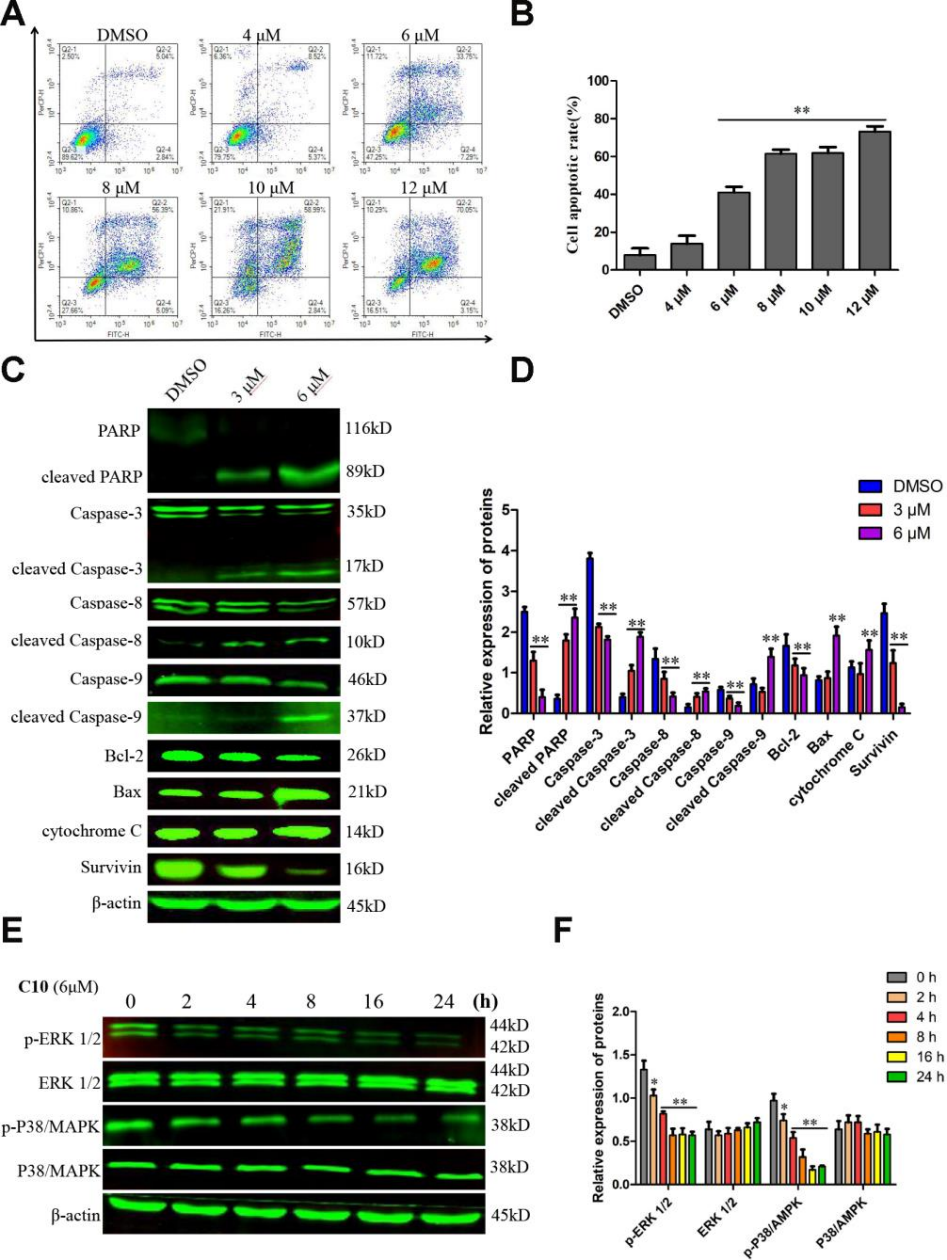
**C10 induced apoptosis in PC3 cells.** (**A**, **B**) PC3 cells were treated with C10 (0, 4, 6, 8, 10 or 12 μM) for 24 h, stained with annexin-V-FITC and PI, and then analyzed by flow cytometry. C10 dose-dependently increased the percentage of annexin-V-FITC-positive apoptotic cells. (**C**, **D**) Western blot showing the expression of PARP, cleaved PARP, Caspase-3, cleaved Caspase-3, Caspase-8, cleaved Caspase-8, Caspase-9, cleaved Caspase-9, Bcl-2, Bax, cytochrome C and Survivin in PC3 cells treated with C10 for 24 h. (**E**, **F**) The phosphorylation levels of core factors in the MAPK signaling pathway (P38/MAPK and ERK1/2) were detected at different time points. β-actin was used as a loading control. Relative expression was determined based on the band intensity compared with that of the loading control. All data shown are representative of three independent experiments. Data are shown as the mean ± SD. *P < 0.05, **P < 0.01 vs. the control group.

To quantify the ability of C10 to induce apoptosis in PC3 cells, we used annexin-V fluorescein isothiocyanate (FITC) and propidium iodide (PI) double staining to analyze the apoptotic rate. Treatment of PC3 cells with increasing concentrations of C10 for 24 h dose-dependently induced apoptosis (Figure 4A and 4B). Importantly, C10 promoted more obvious late apoptosis in the high-dose groups (treated with > 6 μM). We also performed 4',6-diamidino-2-phenylindole (DAPI) staining to visualize the effects of C10 on cell death. Consistently, we observed both nuclear shrinkage and chromatin condensation in C10-treated PC3 cells (Supplementary Figure 2).

Western blot analysis revealed that C10 downregulated the expression of the initiator Caspases (Caspase-8 and Caspase-9) and Caspase-3, but increased the cleavage (activation) of these three Caspases, which thus induced the cleavage of poly ADP ribose polymerase (PARP) (Figure 4C and 4D). The ratio of Bax/Bcl-2 protein also increased remarkably in C10-treated cells. We also investigated the effects of C10 on MAPK pathway members (P38/MAPK and ERK 1/2), which are crucial regulators of apoptosis. The phosphorylation of P38/MAPK and ERK1/2 decreased in a time-dependent manner in C10-treated cells (Figure 4E and 4F).

### Bioinformatics analysis of the correlation between PKCδ and key genes in the secondary pyroptotic pathway

**Figure 5:**
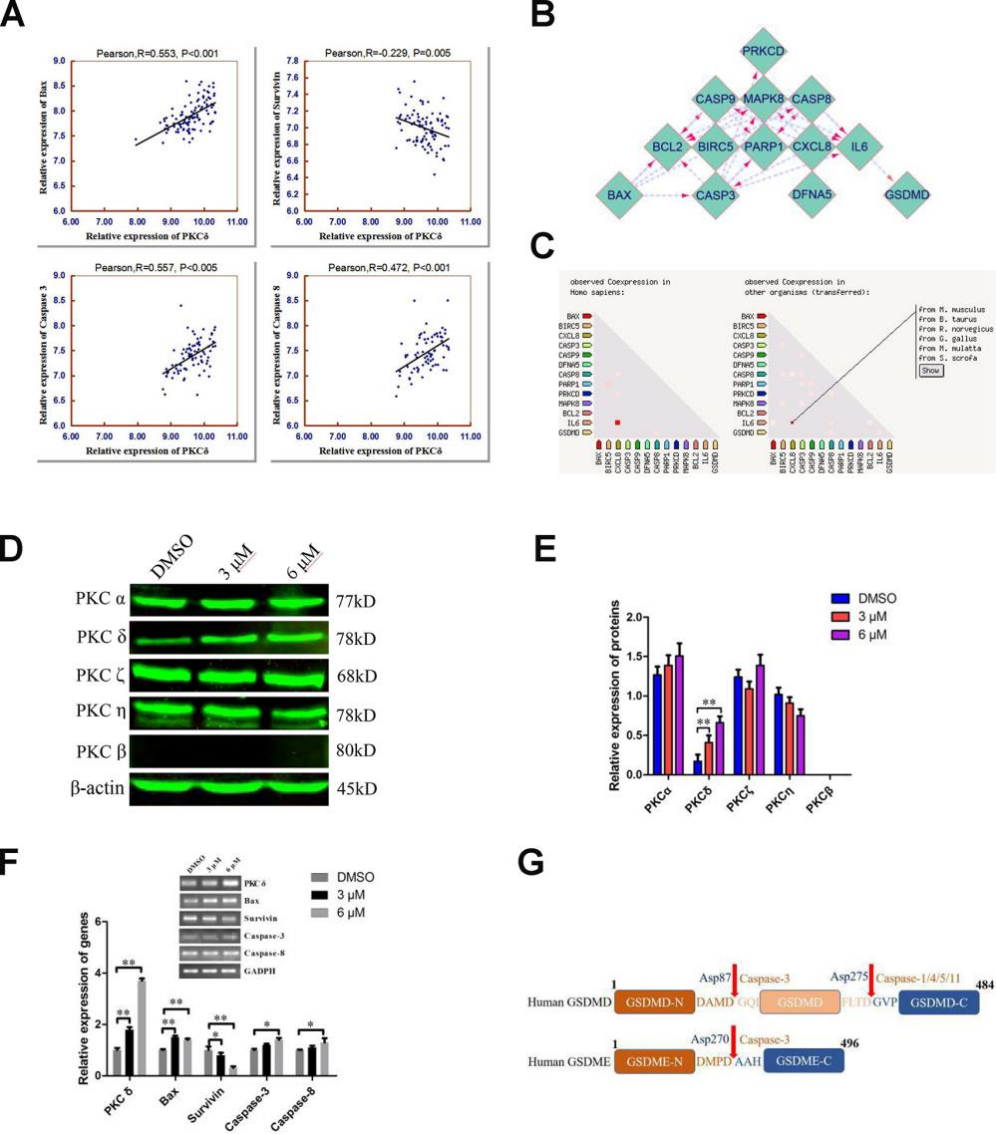
**Combined analyses of the Taylor and STRING databases to predict the correlation between the levels of PKCδ and other core genes in pyroptotic events.** (**A**) Plots of significant Pearson’s correlations between PKCδ levels and Bax, Survivin, Caspase-3 and Caspase-8 levels in the PCa dataset are shown. R is Pearson’s correlation coefficient, and the x and y axes denote the respective genes being analyzed. Data were obtained from the Gene Expression Omnibus. (**B**, **C**) Bioinformatics analysis of PPI and co-expression data in *Homo sapiens* from the STRING database, visualized using Cytoscape 3.7.1. (**D**, **E**) Western blot showing the expression of different PKC subtypes in PC3 cells treated with C10 for 24 h. (**F**) The mRNA levels of PKCδ, Bax, Survivin, Caspase-3 and Caspase-8 were measured by qRT-PCR in PC3 cells treated with C10 for 12 h. All data shown are representative of three independent experiments. Data are shown as the mean ± SD. *P < 0.05, **P < 0.01 vs. the control group. (**G**) Diagrams of the human GSDMD and GSDME proteins. Red arrows indicate the cleavage sites of Caspases.

Our RNA-seq data revealed that C10 treatment altered the expression of a cluster of transcription factors for PCD genes. To further confirm the underlying molecular mechanism of C10-induced PCD in PC3 cells, we performed the bioinformatic analysis. Analysis of 150 PCa cases from the Taylor database revealed that PKCδ mRNA expression correlated positively with Bax, Caspase-3 and Caspase-8 expression, but correlated inversely with Survivin expression (Figure 5A). Then, a protein-protein interaction (PPI) network analysis was performed, and the results were exported and visualized via Cytoscape 3.7.1 (Figure 5B and 5C). PKCδ expression correlated significantly with JNK expression, while JNK expression correlated highly with IL-6 and Bax expression (combined scores of 0.885 and 0.951, respectively).

Next, we analyzed the effects of C10 on the protein levels of different PKC subtypes in PC3 cells (Figure 5D and 5E). Of note, PKCδ was significantly upregulated in C10-treated PC3 cells, whereas there were no obvious changes in other typical isoforms (PKCα, η and ζ), and PKCβ was not detected. Furthermore, the mRNA levels of Bax, Caspase-3, Caspase-8, Survivin and PKCδ were analyzed by qRT-PCR in C10-treated PC3 cells, and the results were consistent with the statistical correlations from the Taylor database (Figure 5F). GSDME is specifically cleaved by Caspase-3 in the linker (Figure 5G), generating a GSDME-N fragment that perforates membrane and induces inflammatory factors release for pyroptosis [5, 20]. Taken together, the C10-treated increases in the mRNA levels of PKCδ, proinflammatory cytokines (e.g., IL-6 and IL-1β) and pro-apoptotic proteins led us to hypothesize that C10 promoted apoptotic events by upregulating PKCδ and then activating Caspase-3 to cleave GSDME, thus generating a necrotic N-terminal fragment capable of inducing pyroptosis.

### C10 induced apoptotic and GSDME-dependent pyroptotic tumor cell death through the PKCδ/JNK signaling pathway

**Figure 6:**
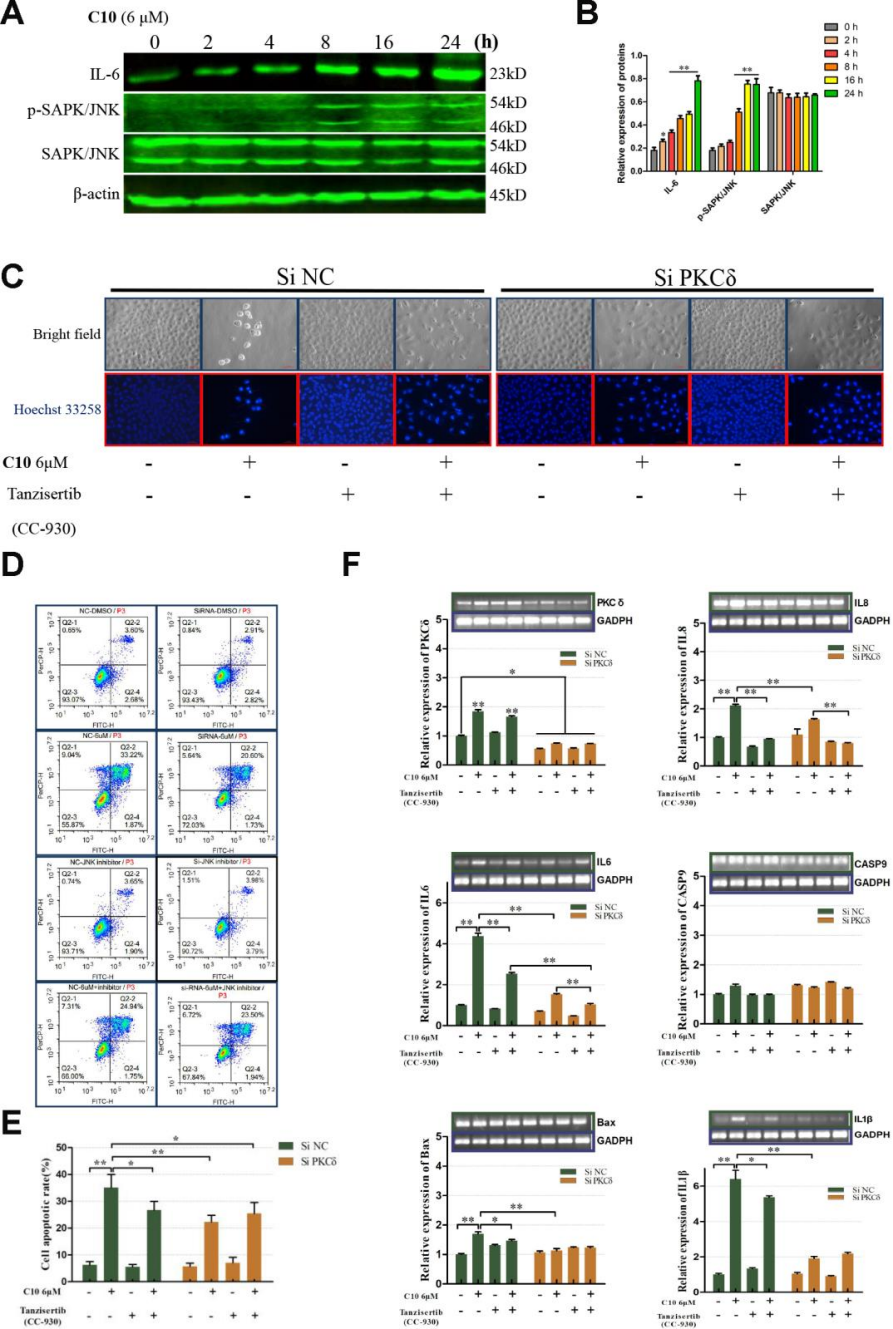
**PKCδ induced PCD by activating JNK signaling in C10-treated PC3 cells.** (**A**, **B**) Western blot of PC3 cells treated for the indicated times (0, 2, 4, 8, 16 or 24 h) with C10. IL-6, p-SAPK/JNK and SAPK/JNK antibodies were used. (**C**) Cultured PC3 cells were treated with C10 in the presence of different inhibitors (siPKCδ and the JNK-specific inhibitor Tanzisertib [CC-930]) for 24 h. The cells were then stained with Hoechst 33258 and photographed using a fluorescence microscope (magnification ×200, scale bar: 100 μm). (**D**, **E**) Cultured PC3 cells were stained with annexin-V-FITC and PI for flow cytometry analysis. (**F**) The mRNA levels of PKCδ, Caspase-9, IL-6, IL-8, IL-1β and Bax were measured by qRT-PCR. All data shown are representative of three independent experiments. Data are shown as the mean ± SD. *P < 0.05, **P < 0.01 vs. the control group.

The release of inflammatory cytokines is an essential characteristic of pyroptosis. Among the many known inflammasome complexes, the NLR pyrin domain containing 3 (NLRP3) and pro-Caspase-1 complexes are the best characterized [17, 19]. To estimate the effects of C10 on inflammasome pathways, we measured the protein levels of core inflammatory cytokines such as NLRP3, IL-6, phosphorylated nuclear factor kappa B (p-NF-κB), p-JNK and Caspase-1 (Figure 6A and 6B, Supplementary Figure 3B). Notably, the proteins NLRP3, p-NF-κB and Caspase-1 were not detected in C10-treated PC3 cells, and even their mRNA levels were extremely low (qRT-PCR data not shown), consistent with the RNA-Seq results. On the contrary, IL-6 expression and JNK phosphorylation were upregulated in a time-dependent manner following C10 treatment.

Given the PPI network analysis results and the diverse functions of PKCδ, we wanted to confirm the involvement of PKCδ in apoptotic and GSDME-dependent pyroptotic tumor cell death. Thus, we treated PC3 cells with small interfering RNA (siRNA) against PKCδ (siPKCδ, Supplementary Figure 3A) or with the JNK-specific inhibitor Tanzisertib (CC-930). Flow cytometry and Hoechst 33258 staining assays revealed that siPKCδ and Tanzisertib (CC-930) treatment each significantly diminished C10-induced apoptosis (Figure 6C–6E). We also examined the mRNA levels of PKCδ and various core genes after treating PC3 cells with C10 and siPKCδ or Tanzisertib (CC-930). The mRNA levels of IL-6, IL-8, IL-1β and Bax decreased when the cells were co-incubated with C10 and siPKCδ or Tanzisertib (CC-930), while the mRNA expression of Caspase-9 did not evidently change (Figure 6F).

**Figure 7:**
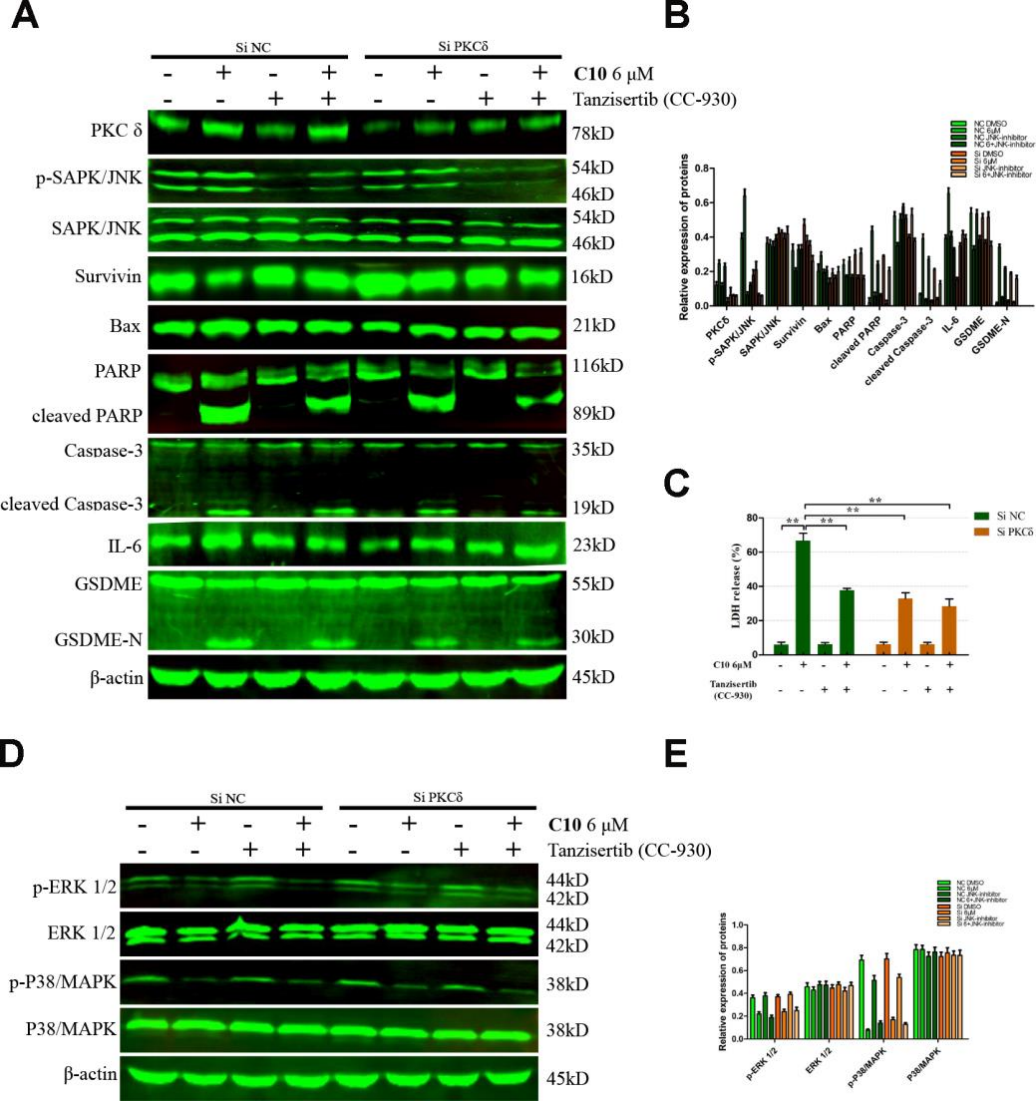
**Inhibition of PKCδ suppressed C10-induced concurrent apoptosis and GSDME-dependent pyroptosis in PC3 cells.** (**A**, **B**) Cultured PC3 cells were incubated with C10 in the presence of siPKCδ or the JNK-specific inhibitor Tanzisertib (CC-930) for 24 h to determine the link between the apoptotic and pyroptotic pathways. The protein levels of PKCδ, p-SAPK/JNK, SAPK/JNK, Survivin, Bax, PARP, cleaved PARP, Caspase-3, cleaved Caspase-3, IL-6, GSDME and GSDME-N were examined by Western blotting. (**C**) LDH enzyme activity was measured in the culture supernatants of PC3 cells after various treatments. (**D**, **E**) The phosphorylation levels of core factors in the MAPK signaling pathway (P38/MAPK and ERK1/2) were detected after various treatments in PC3 cells. β-actin was used as a loading control. All data shown are representative of three independent experiments. Data are shown as the mean ± SD. *P < 0.05, **P < 0.01 vs. the control group.

**Figure 8:**
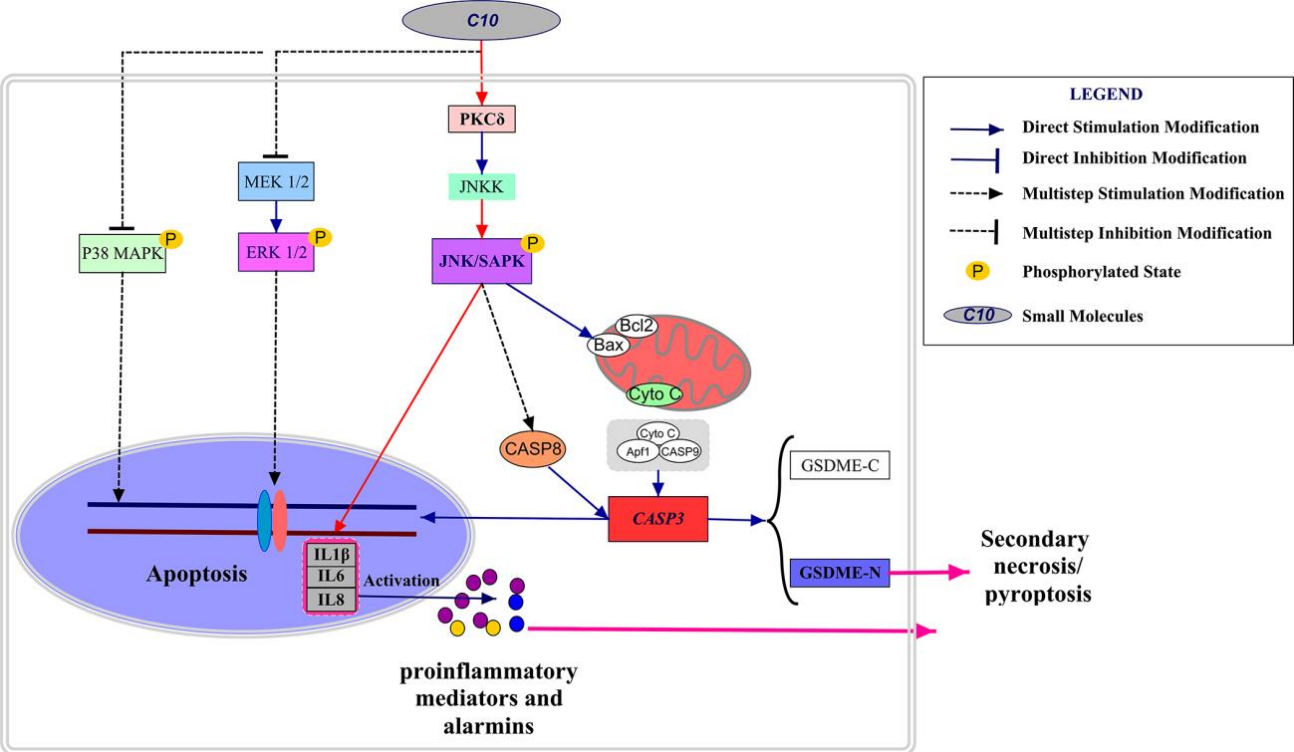
**Schematic diagram depicting the anti-PCa mechanism of C10.** C10 stimulated the PKCδ/JNK/IL-6 signaling pathway and thus induced crosstalk between apoptosis and GSDME-dependent pyroptosis in PC3 cells. Red arrows: the new signal transduction pathway discovered in our study, whereby PKCδ/JNK/IL-6 lead to concurrent apoptosis and pyroptosis.

We then performed Western blotting on the PC3 cells, and found that both siPKCδ and Tanzisertib (CC-930) significantly attenuated C10-induced secondary necrosis/pyroptosis by upregulating Survivin, downregulating Bax and IL-6, and inhibiting the activation of Caspase-3 and GSDME (Figure 7). Interestingly, the phosphorylation of JNK decreased in PC3 cells co-treated with C10 and siPKCδ, indicating that PKCδ may be an upstream inducer of the JNK/IL-6 pathway (Figure 7A and 7B). The release of lactate dehydrogenase (LDH) indicated that C10 treatment destroyed the integrity of the PC3 cell membrane. Of note, both siPKCδ and Tanzisertib (CC-930) markedly inhibited C10-induced LDH release (Figure 7C). Furthermore, the effects of C10 on P38/MAPK and ERK1/2 were not enhanced by siPKCδ or Tanzisertib (Figure 7D and 7E). These results suggested that the inhibition of PKCδ/JNK signaling suppressed C10-induced apoptotic and GSDME-dependent pyroptotic cell death (Figure 8).

### C10 inhibited PCa tumor growth in a xenograft model *in vivo*

**Figure 9:**
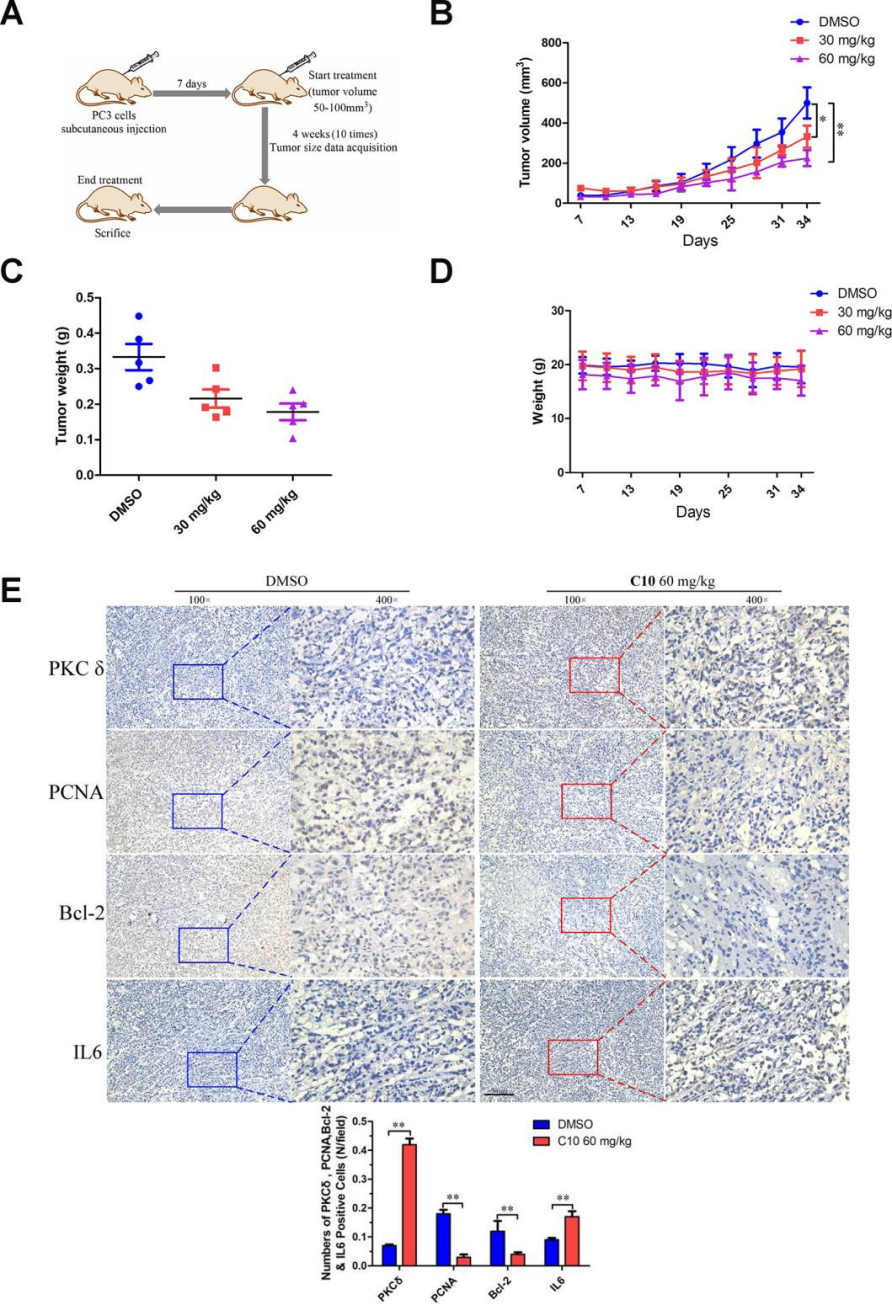
**C10 attenuated tumor growth by inhibiting cell proliferation and inducing apoptosis and inflammation in PC3 xenograft mice.** (**A**) PC3 cell tumor xenograft nude mice were intraperitoneally administered C10 (low-dose or high-dose) or the control treatment every two days for a total of 10 times, as indicated in the diagram. (**B**) The tumor sizes in the three groups were monitored and recorded at three-day intervals as soon as C10 was injected. (**C**) The subcutaneous tumors were weighed immediately at the end of the study. (**D**) The mouse weights in the three groups were recorded at three-day intervals as soon as C10 was injected. (**E**) Tumor spheroids generated from the control and high-dose groups were fixed, sectioned and immunohistochemically stained for PKCδ, PCNA, Bcl-2 and IL-6 expression. The levels of the indicated proteins were quantified in the control and high-dose groups (Scale bar: 50 μm). Data are shown as the mean ± SD. *P < 0.05, **P < 0.01.

We then used a PC3 cell xenograft tumor model to determine whether C10 could suppress PC3 tumor growth and upregulate PKCδ expression *in vivo*. Initially, mice were intraperitoneally injected with 30 or 60 mg/kg of C10 every two days (ten times in total) (Figure 9A). Tumor growth was significantly lower in the C10-treated group than in the control group after 25 days (Figure 9B and 9C). Body weight was also measured as an indicator of general health, and no obvious decrease was found in any group (Figure 9D). Moreover, immunohistochemistry analysis revealed that PKCδ expression and IL-6 secretion were remarkably elevated in the C10-treated group, in agreement with the *in vitro* results. We also examined the proliferation marker PCNA and the pro-apoptotic protein Bcl-2 to assess the effects of C10 treatment on the proliferation and apoptosis of the tumor xenografts (Figure 9E). The results demonstrated that C10 significantly reduced the proliferation of PCa *in vivo*.

## DISCUSSION

Modern drug discoveries have demonstrated that new drug candidates can be developed more easily when the lead molecules are natural products rather than compounds derived from synthetic or combinatorial structures [31]. Approximately 40% of all drugs in the world are directly or indirectly derived from natural products, such as artemisinin, a notable success story of a drug discovered from traditional Chinese medicine [32, 33]. Chalcones containing geranyl or prenyl groups are the most structurally diverse subclass of flavonoids. Chalcones are widely found in nature, and have drawn a great deal of attention due to their biological and pharmacological activities [30, 34]. In this study, we found that a novel 3’,5’-diprenylated chalcone (C10) could effectively inhibit the proliferation of PC3 cells *in vitro* and *in vivo*.

From an evolutionary perspective, cancer is an adaptive and complex system, so the expected effects of anti-cancer therapies have become increasingly difficult to achieve through the prolonged use of single-target drugs [35, 36]. To overcome or prevent acquired resistance, we are committed to rationally designing multi-target drugs that can inhibit more than one of the cellular signaling pathways hijacked by cancer cells. Recently, anlotinib hydrochloride, a new orally administered tyrosine kinase inhibitor, was found to have extensive advantages in treating non-small cell lung cancer by targeting KIT, VEGFA and FGFR1 [37]. In this study, we demonstrated that C10 suppressed the growth and progression of PC3 cells by perturbing multiple signaling pathways. Cell viability assays revealed that C10 more strongly inhibited the proliferation of PC3 cells than of DU145 and RWPE-1 cells at the same doses and time points. Moreover, by analyzing KEGG enrichment pathways and performing a series of pharmacological assays, we discovered that C10 exerted these effects by altering the expression of core proteins associated with cell cycle arrest, apoptosis and pyroptosis, reflecting its suitability for multi-target and multi-pathway therapy.

In our RNA-seq analyses, the C10-induced increases in PKCδ and proinflammatory cytokine mRNA levels aroused our attention. Proteins in the PKC family of serine/threonine kinases are activated by diverse stimuli, and function as upstream regulators of many biological processes [22]. The different activation mechanisms of PKCα, ε and δ, which result from the different structural and functional properties of their C1 domains, have been best characterized in various cancer cells [38, 39]. Regarding PCa, numerous studies have indicated that PKCδ promotes tumor cell invasion and migration, while others have demonstrated that PKCδ upregulation is required for death receptor-induced apoptosis [40, 41].

To determine the effects of C10 on Caspase-dependent apoptotic events in PC3 cells, we investigated the expression profiles of core pro-apoptotic proteins. We observed that cleaved Caspase-3, -8 and -9 levels and cleaved PARP levels increased in C10-treated cells, as did the Bax/Bcl-2 ratio. Interestingly, C10 promoted apoptotic progression through the MAPK pathway by inducing JNK phosphorylation and P38 MAPK/ERK dephosphorylation, as previously reported [8]. Given the PPI network and gene correlation analysis results, we further hypothesized that PKCδ directly phosphorylated JNK, which thus transduced apoptotic signals. As expected, the inhibition of PKCδ suppressed the expression of Bax, cleaved Caspase-3 and cleaved PARP. In addition, siPKCδ significantly attenuated JNK phosphorylation in C10-treated cells, without altering P38 MAPK or ERK1/2 phosphorylation. Thus, PKCδ may have promoted Caspase-dependent apoptosis by activating JNK signaling in C10-treated PCa cells.

The molecular regulation of PCD has been widely used to treat a variety of human cancers [42]. Over the past decade, the mechanisms of apoptosis, necrosis, necroptosis and pyroptosis have been extensively investigated [18, 43, 44]. However, little is known about the potentially important crosstalk between apoptotic and pyroptotic processes following pharmacological interventions. Many studies have demonstrated that inflammatory Caspases such as the canonical Caspase-1 cleave GSDMD to trigger secondary pyroptotic cell death [20]. Therefore, we assessed the protein levels of core inflammatory cytokines including NLRP3, p-NF-κB and Caspase-1 in PC3 cells (Supplementary Figure 3B). Notably, NLRP3, p-NF-κB and Caspase-1 were not detected at the protein level, and their mRNA levels were extremely low (qRT-PCR data not shown).

Previous studies have demonstrated that GSDME, the other member of the gasdermin superfamily, possesses the same pore-forming function as GSDMD, and can be cleaved by Caspase-3 after Asp270 [5]. Wang et al. reported that activated Caspase-3 triggered pyroptosis by cleaving GSDME, illustrating a non-canonical form of pyroptosis that offered new insights into cancer chemotherapy [20]. The current study preliminarily indicated that C10 induced apoptotic events by activating the PKCδ/JNK pathway, which stimulated Caspase-3 activation and GSDME cleavage, ultimately generating a necrotic N-terminal GSDME fragment capable of inducing pyroptosis and inflammatory cytokine release. To verify that C10 treatment induced apoptosis and non-canonical pyroptosis through the PKCδ/JNK pathway, we treated PC3 cells with siPKCδ or the JNK-specific inhibitor Tanzisertib (CC-930). Both siPKCδ and Tanzisertib (CC-930) significantly attenuated C10-induced secondary necrosis/pyroptosis by inhibiting GSDME activation and downregulating inflammatory complex members such as IL-6, IL-8 and IL-1β. Of note, both siPKCδ and Tanzisertib (CC-930) also markedly inhibited C10-induced LDH release, indicating that inhibiting PKCδ and JNK significantly reduced the C10-induced damage to the cell membrane integrity. Thus, we demonstrated that C10 could induce apoptosis and GSDME-dependent pyroptosis through the PKCδ/JNK pathway. It was suggested that there might exist the crosstalk between apoptosis and GSDME-dependent pyroptosis in the PC3 cells treated with C10. However, the precise mechanism for this crosstalk, including the function and expression of GSDME, remains to be further investigated.

This study (summarized in Figure 8) has challenged the traditional view that Caspase-dependent apoptosis is the sole death route for molecularly targeted therapies. It might indicate that the potential clinical relevance of GSDME expression and pyroptosis in PCa, and demonstrated the crosstalk between C10-induced apoptosis and pyroptosis through the PKCδ/JNK pathway.

## MATERIALS AND METHODS

### Reagents

The PKCδ-specific siRNA and negative-control siRNA were designed and synthesized by GenePharma (Shanghai, China), and Lipofectamine^TM^ 3000 Reagent was purchased from Thermo Fisher. Tanzisertib (CC-930) was purchased from Sellectchem (Sellectchem, Houston, TX, USA) and dissolved in DMSO. Primary antibodies against c-Myc, CDK2, CDK6, Cyclin D2, P21^cip1^, P27^kip1^, PCNA, cytochrome C, PKCδ, PKCα, PKCη, PKCβ, PKCζ, IL-6, ERK1/2, p-ERK1/2, P38/MAPK, p-P38/MAPK and GSDME were obtained from Abcam (Cambridge, UK). Antibodies against Caspase-3, Caspase-8, cleaved Caspase-8, Caspase-9, cleaved Caspase-9, PARP, Bax, Bcl-2, Cyclin D1, Survivin, SAPK/JNK, p-SAPK/JNK, NF-κB/P65, p-NF-κB/P65, Caspase-1 and NLRP3, along with anti-mouse and anti-rabbit secondary antibodies, were purchased from Cell Signaling Technology (Beverly, MA, USA). FastStart Universal SYBR Green Master (Rox) was purchased from Sigma-Aldrich (Roche, Germany). Primers were prepared by Invitrogen (Shanghai, China). Other reagents were analytical-grade or guaranteed reagent commercial products, and were used without further purification, unless otherwise noted.

### Cell culture

Human non-tumorigenic (RWPE-1) and tumorigenic (DU145 and PC3) prostate cell lines were gifts from the Sunnybrook Research Center (Toronto, ON, Canada). Cells were cultured in DMEM (Hyclone, South Logan, UT, USA) supplemented with 10% FBS, 100 U/mL penicillin and 100 μg/mL streptomycin (Sijiqing, Hangzhou, China) at 37 °C in a CO_2_ incubator (5% CO_2_ and 95% air; 95% humidity). Cells were passaged at least three times before being used for cell-based assays.

**Cytotoxicity assay**

Cytotoxicity was measured with the MTT assay. Cells (6×10^3^) were seeded into 96-well plates, incubated at 37 °C for 24 h, and then treated with the test compound (C10) for the indicated time (24, 48 or 72 h). After this time, 20 μL of MTT solution (5 mg/mL) was added to each well, and the plates were incubated for 4 h at 37 °C. The resulting formazan crystals were dissolved in 150 μL of DMSO and quantified on a microplate reader (Vario Skan Flash, Thermo Scientific, USA) at 490 nm.

### Clonogenic survival assay

PC3 and DU145 cells were plated in medium plates at a confluent density of 3×10^5^ cells/plate, and then were treated with various concentrations of C10 for 24 h. The cells were trypsinized, resuspended in the medium and counted. Then, the cells were re-seeded (1000 cells per medium plate) and incubated for 14 days. Fresh medium was added every four days. After that, the cells were fixed with 4% (w/v) paraformaldehyde for 15 min and stained with 0.1% (w/v) crystal violet for 10 min.

### Cell cycle and apoptosis assay

The cell cycle was assessed by flow cytometry using PI (1 mg/mL) (Solarbio, Beijing, China), and apoptosis was assessed by flow cytometry using a staining kit containing annexin-V-FITC and PI (BD Pharmingen, San Diego, CA, USA). PC3 cells (3×10^5^) were seeded in six-well plates for 24 h and then incubated with C10 or the vehicle (DMSO) for 24 h. Then, the cells were trypsinized and washed twice with cold PBS. For the cell cycle analysis, the cells were fixed with cold 75% ethanol overnight at -20 °C. After being centrifuged at 1000 rpm for 5 min, the cells were washed with cold phosphate-buffered saline, stained with PI for 30 min at 37 °C, and analyzed on a flow cytometer. For the apoptosis experiment, the cells were resuspended in 1× binding buffer, incubated with 5 μL of PI and FITC for 15 min at room temperature in the dark, and analyzed on a flow cytometer (Becton Dickinson, Franklin Lakes, NJ, USA).

### Hoechst 33258 and DAPI staining assay

The Hoechst 33258 and DAPI staining assay has been previously described [8]. Briefly, cells were seeded in a six-well plate at an initial density of 3×10^5^ cells per well. The cells were then treated with different concentrations of C10 for 24 h, stained with Hoechst 33258 and DAPI (Beyotime, Jiangsu, China) in accordance with the kit guidelines, and photographed under a fluorescence microscope with a camera (Nikon Corporation, Japan).

### LDH release assay

Pyroptosis and necrosis were quantified based on the release of LDH into the cell culture medium after various treatments. An LDH cytotoxicity assay kit (Beyotime Biotech) was used according to the manufacturer’s instructions. After the cells were incubated with the kit reagents at room temperature for 30 min, the absorbance was read at a wavelength of 490 nm. The LDH activity in the culture supernatant was calculated as a percentage of the total LDH in the cell lysate.

### qRT-PCR

After cells had been treated with C10 for 12 h, qRT-PCR was performed on a StepOne Plus thermal cycler (Applied Biosystems, Forest City, CA, USA) using specific primers and FastStart Universal SYBR^®^ Green Select Master Mix (Roche, IN, USA) as previously described [8]. The GAPDH gene was used as a control. The PCR products were subjected to electrophoresis on a 1% agarose gel, and were photographed using a Gel Documentation System (Syngene, England). The primer sequences are shown in Supplementary Table 2.

### Western blotting

Western blotting was conducted as previously described [8]. Equal amounts of cell lysates (80-100 μg) were separated on a 6-10% sodium dodecyl sulfate polyacrylamide gel and transferred to a polyvinylidene difluoride membrane. An Odyssey Infrared Imaging System was used to detect and visualize the immunoreactive proteins. The experiments were performed at least three times, and densitometric analysis was performed with Image-pro Plus 6.0 software. β-actin was used as a control.

### RNA extraction and sequencing

PC3 cells were treated with C10 for 12 h and then subjected to RNA sequencing. Total RNA was extracted with TRIzol reagent (Invitrogen, USA) based on the manufacturer’s instructions. The cDNA library was prepared and sequenced by Shanghai Personal Biotechnology Co., Ltd (Shanghai, China) on an Illumina NextSeq 500 platform. We used DESeq (version 1.18.0) to identify differentially expressed genes, which were screened based on transcriptional |fold change| values > 2 and *P*-values < 0.05. The dysregulated genes were redacted using the R package (ggplots2 software) and visualized as a volcano plot. KEGG pathway enrichment analyses were performed to categorize the considerably enriched functional classifications or metabolic pathways of the differentially expressed genes. To compare the differences in differentially expressed gene profiles between two samples, we clustered the gene expression data using the heatmap software package.

### siRNA

Cells were seeded at a density of 5×10^5^ cells/dish in 60-mm dishes, and grown to 50% confluent density at the time of transfection. Then, 50 nM PKCδ-specific siRNA and negative-control siRNA were simultaneously transfected into the cells using the Lipofectamine^TM^ 3000 Reagent according to the manufacturer’s instructions. The cells were incubated at 37 °C for two days, and then were subjected to different treatments. The sequences of siPKCδ and the negative-control siRNA are shown in Supplementary Table 3.

### *In vivo* tumorigenic assay and immunohistochemistry

The animal experiment was approved by the Institutional Animal Care and Use Committee at Guangzhou University of Chinese Medicine. BALB/c nude mice (four- to five-week old males) were randomly allocated into three groups of five mice each. PC3 cells (5×10^6^ cells in an equal volume of Matrigel) were subcutaneously injected into the flank of each nude mouse. After two weeks of tumor growth, the mice were intraperitoneally administered C10 (30 or 60 mg/kg) every two days for ten times in total, and DMSO-injected mice were used as the control group. Tumor size and weight were measured at three-day intervals as soon as C10 was injected, and the tumor volumes were calculated as V (mm^3^)=width^2^ (mm^2^) × length (mm) / 2.

After treatment, the mice were humanely sacrificed by cervical dislocation under anesthesia. Tumor tissues were fixed in 4% paraformaldehyde for 24 h at room temperature, and then were embedded in paraffin. The paraffin-embedded tissues were subjected to immunohistochemistry analyses with antibodies against PCNA, PKCδ, Bcl-2 and IL-6 using a 3, 3’-diaminobenzidine substrate kit (Solarbio, Beijing, China) in accordance with the manufacturer’s protocol. Slides were photographed under a light microscope (Nikon Corporation, Japan).

### Bioinformatics analysis

The PPIs of the differentially expressed genes were evaluated in the STRING database (https://string-db.org/). Cytoscape 3.7.1 was used to visualize the PPI network. To investigate the correlation between the mRNA levels of PKCδ and key genes in cell death signaling pathways, we analyzed clinical information from 150 PCa tissues and 29 adjacent non-cancerous prostate tissues in the Taylor database.

### Statistical analysis

All experiments were repeated at least three times, and the results are expressed as the mean ± standard deviation (SD). Statistical analyses were performed with Student’s t-test or one-way analysis of variance, and *P* < 0.05 was considered statistically significant. *P < 0.05, **P < 0.01.

## Supplementary Material

Supplementary Figures

Supplementary Tables
