# Supplementary material for: A novel 3’,5’-diprenylated chalcone induces concurrent apoptosis and GSDME-dependent pyroptosis through activating PKCδ/JNK signal in prostate cancer
Source: Aging (Albany NY). 2020 May 19;12(10):9103–24. doi: 10.18632/aging.103178 (PMC7288973; doi:10.18632/aging.103178)
Supplement: Supplementary Figures [file aging-12-103178-s001..pdf]

SUPPLEMENTARY FIGURES

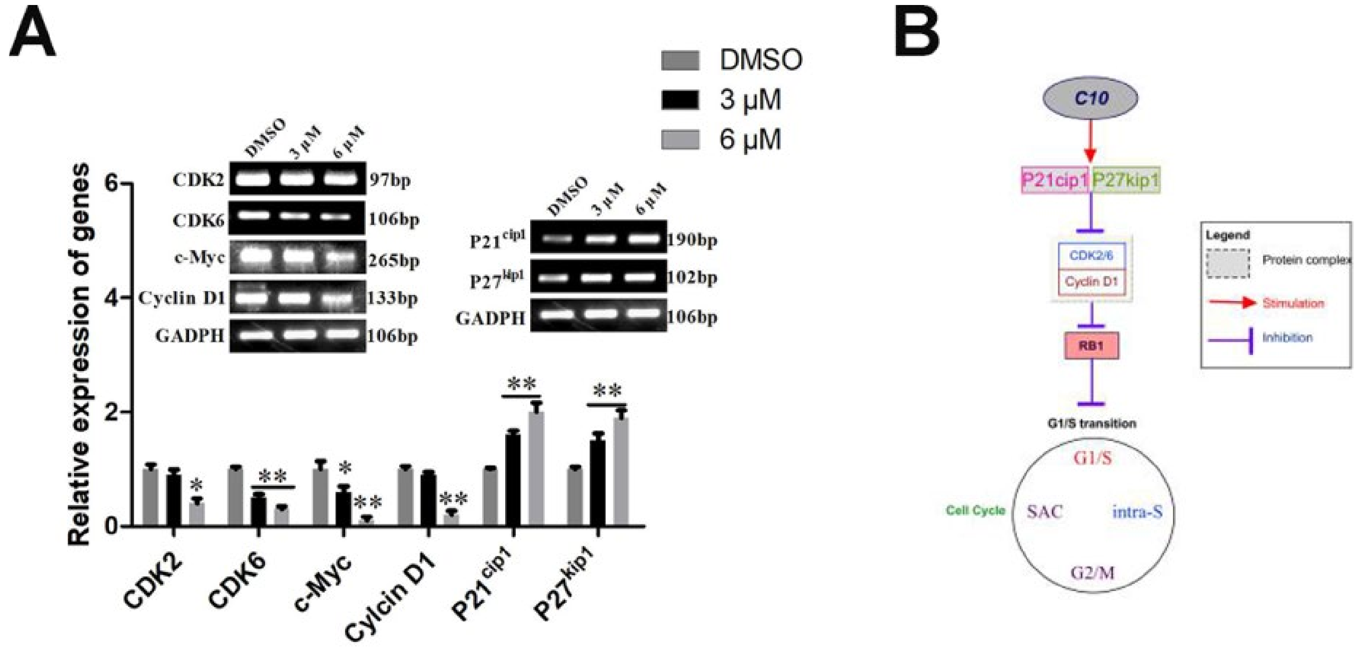

Supplementary Figure 1. C10 induced sub-G1 phase cell cycle arrest in cultured PC3 cells. (**A**) PC3 cells were exposed to C10 (3 or 6 μM) for 12 h, and core genes associated with the cell cycle, including CDK2, CDK6, c-Myc, Cyclin D1, $P21^{cip1}$ and $P27^{kip1}$ were measured by qRT-PCR. (**B**) Model depicting how C10 induced cell cycle arrest in sub-G1 phase by upregulating $P21^{cip1}$ and $P27^{kip1}$ and then downregulating CDKs and Cyclins. All data shown are representative of three independent experiments. *$P < 0.05$, **$P < 0.01$ vs. the control group.

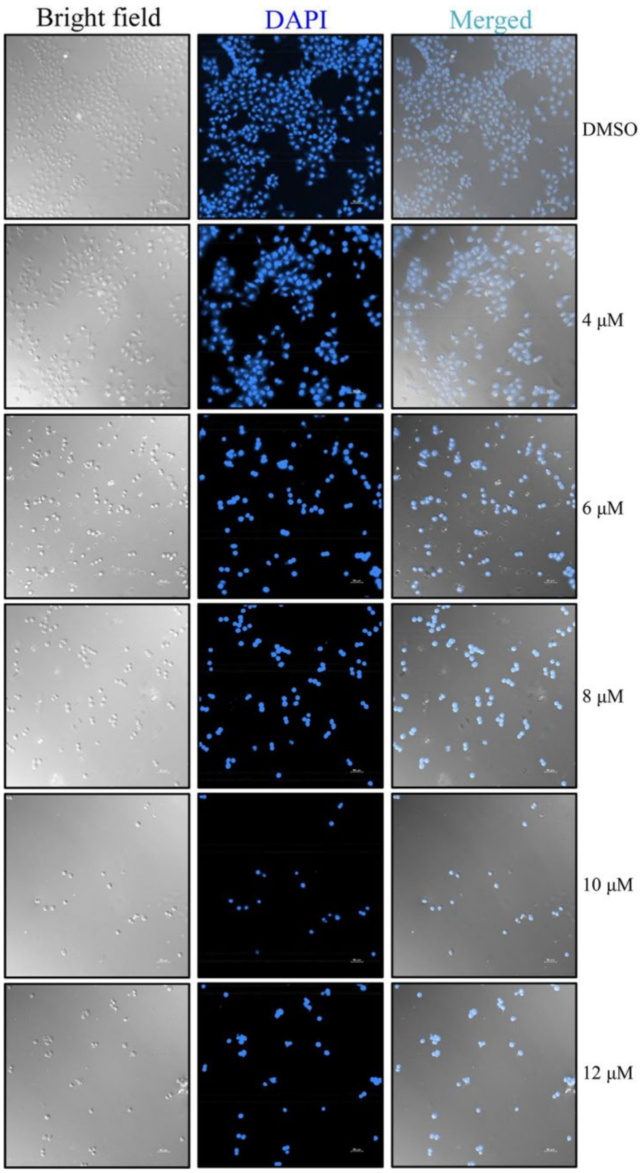

Supplementary Figure 2. C10 induced apoptosis in PC3 cells. PC3 cells were treated with the indicated concentrations of C10 (0, 4, 6, 8, 10 or 12 μM) for 24 h and then photographed using a fluorescence microscope. Images depict DAPI staining in the different groups (left, bright field; middle, DAPI staining; right, merged). Scale bar: 50 μm.

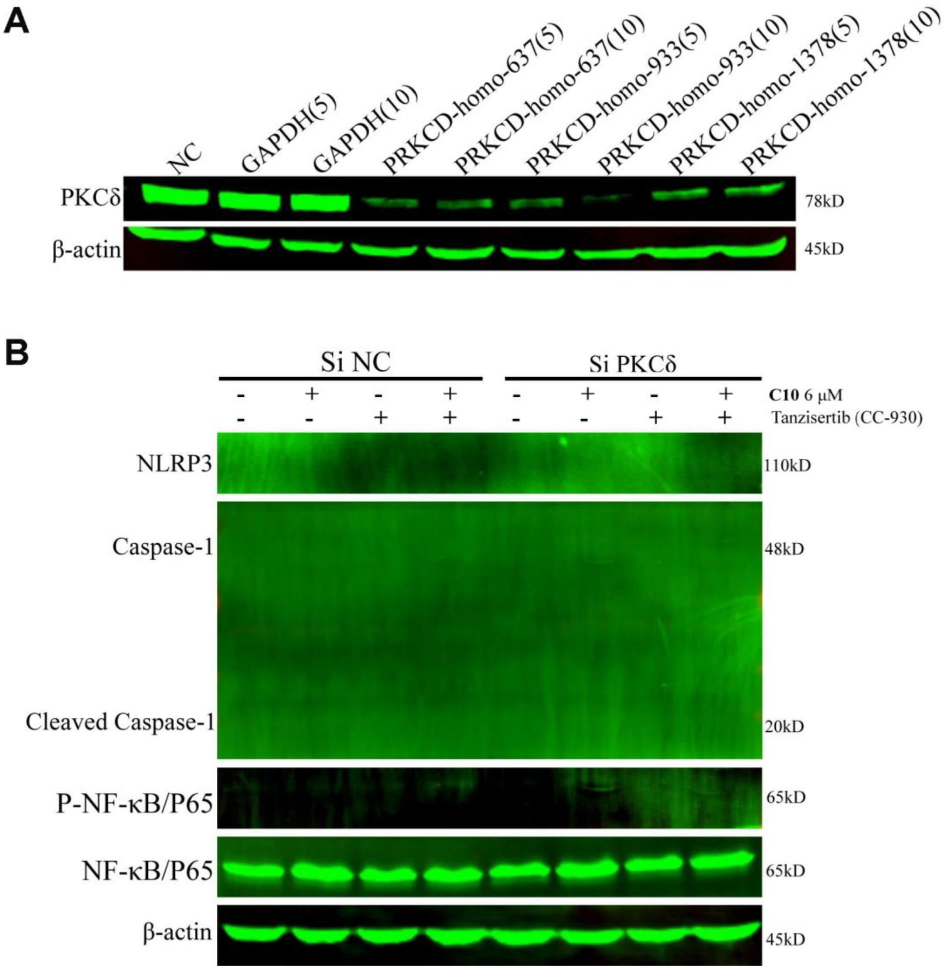

Supplementary Figure 3. Effects of C10 on the canonical inflammatory Caspase-1 signaling pathway. (**A**) PKCδ protein expression was detected in PC3 cells 48 h after transfection with siPKCδ, negative control siRNA or GAPDH. (**B**) Cultured PC3 cells were incubated with C10 in the presence of siPKCδ or the JNK-specific inhibitor Tanzisertib (CC-930) for 24 h. Then, the protein levels of NLRP3, Caspase-1, cleaved Caspase-1, p-NF-κB/P65 and NF-κB/P65 were detected by Western blotting. β-actin was used as a loading control.
