# Supplementary material for: A novel 3’,5’-diprenylated chalcone induces concurrent apoptosis and GSDME-dependent pyroptosis through activating PKCδ/JNK signal in prostate cancer
Source: Aging (Albany NY). 2020 May 19;12(10):9103–24. doi: 10.18632/aging.103178 (PMC7288973; doi:10.18632/aging.103178)
Supplement: Supplementary Tables [file aging-12-103178-s002..pdf]

SUPPLEMENTARY TABLES

Supplementary Table 1. Enrichment of signaling pathways most relevant to PC3 cell progression.

Pathway	Up-regulated Genes	Down-regulated Genes	Total Numbers	P-Value
PI3K-AKT signaling path way	3	8	340	0.002558006
MAPK signaling pathway	5	4	253	0.0033273
NOD-like receptor signaling pathway	3	0	55	0.02749174
Apoptosis	3	1	140	0.08547306

Supplementary Table 2. Primer sequences used in this study.

Genes	Primer	Primer Sequence (5'→3')
PKCδ	Forward	CCGCTTTGAACTCTACCGTG
	Reverse	TTTGCACATCCCAAAGTCGG
Caspase-8	Forward	GGGACAGGAATGGAACACACTTGG
	Reverse	TCAGGATGGTGAGAATATCATCGCC
Caspase-3	Forward	GAAATTGTGGAATTGATGCGTGA
	Reverse	CTACAACGATCCCCTCTGAAAAA
c-Myc	Forward	TCCTGGCAAAAGGTCAGAGT
	Reverse	GTTGTGTGTTCGCCTCTTGA
Bax	Forward	CCCGAGAGGTCTTTTTCCGAG
	Reverse	CCAGCCCATGATGGTTCTGAT
Survivin	Forward	CCACTGAGAACGAGCCAGACTT
	Reverse	GTATTACAGGCGTAAGCCACCG
Caspase-9	Forward	CTCAGACCAGAGATTCGCAAAC
	Reverse	GCATTTCCCCTCAAACTCTCAA
IL-6	Forward	ACTCACCTCTTCAGAACGAATTG
	Reverse	CCATCTTTGGAAGGTTCAGGTTG
IL-8	Forward	GAGAGTGATTGAGAGTGGACCAC
	Reverse	CACAACCCTCTGCACCCAGTTT
IL-1β	Forward	CCACAGACCTTCCAGGAGAATG
	Reverse	GTGCAGTTCAGTGATCGTACAGG
$P21^{cip1}$	Forward	GGTGGCAGTAGAGGCTATGG
	Reverse	GCCGAGAGAAAACAGTCCAG
$P27^{kip1}$	Forward	CAAGGAAGGTTCATGTAGAGAAAAG
	Reverse	CAAATGGTTTTTCCATACACAGG
CDK2	Forward	ATGGATGCCTCTGCTCTCACTG
	Reverse	CCCGATGAGAATGGCAGAAAGC
CDK6	Forward	GGATAAAGTTCCAGAGCCTGGAG
	Reverse	GCGATGCACTACTCGGTGTGAA
Cyclin D1	Forward	TCTACACCGACAACTCCATCCG
	Reverse	TCTGGCATTTTGGAGAGGAAGTG
GAPDH	Forward	GCAAATTCCATGGCACCGT
	Reverse	TCGCCCCACTTGATTTTGGAGG

Supplementary Table 3. The sequences of siPKCδ and the negative control siRNA.

PKCδ-637-sense	GCAAGAAGAACAAUGGCAATT
PKCδ-637-antisense	UUGCCAUUGUUCUUCUUGCTT
PKCδ-933-sense	GCUGCCAUCCACAAGAAAUTT
PKCδ-933-antisense	AUUUCUUGUGGAUGGCAGCTT
PKCδ-1378-sense	GCAAGUGCAACAUCAACAATT
PKCδ-1378-antisense	UUGUUGAUGUUGCACUUGCTT
negative control	UUCUCCGAACGUGUCACGUTT
negative control	ACGUGACACGUUCGGAGAATT
